# Phenolic Characterization and Quality Evaluation of Herbal Coffee from Roasted Juniper Berry Fruits (*Juniperus drupacea* L.): Elucidating the Impact of Roasting

**DOI:** 10.3390/foods13233946

**Published:** 2024-12-06

**Authors:** Hasim Kelebek, Merve Carikcioglu, Pınar Kadiroglu, Esra Ereli, Turkan Uzlasir, Serkan Selli

**Affiliations:** 1 Department of Food Engineering, Faculty of Engineering, Adana Alparslan Turkes Science and Technology University, 01250 Adana, Türkiyepkadiroglu@atu.edu.tr (P.K.); uzlasirturkan@gmail.com (T.U.); 2 Vocational School of Technical Sciences, Food Technology, Mersin University, 33100 Mersin, Türkiye; 3 Department of Food Engineering, Faculty of Agriculture, Cukurova University, 01330 Adana, Türkiye; sselli@cu.edu.tr

**Keywords:** juniper berry, *Juniperus drupacea*, roasting, herbal coffee, antioxidant activity, phenolic compounds

## Abstract

Consumers’ demand for foods with health benefits and different tastes is on an increasing trend. Juniper berries (“andiz” in Turkish) are the fruits of perennial, aromatic, and resinous *Juniperus drupacea* trees. In this study, quality properties of herbal coffee samples obtained from juniper berries roasted at three different temperatures (120, 160, and 200 °C) and four different durations (10, 25, 32.5, and 55 min) were elucidated. The herbal coffee samples were prepared from roasted and powdered fruits, and their total phenolic contents (TPCs), sugar profiles, antioxidant activities (AAs), and other quality parameters were examined. The highest AA value was determined as 17.99 and 29.36 mM Trolox/L (DPPH and ABTS, respectively) in the herbal coffee prepared from berries roasted at 120 °C for 25 min. Sucrose and glucose were dominant in all herbal coffee samples. Sixteen phenolic compounds were identified and quantified by a LC-ESI-MS/MS device. The TPC values of the herbal coffee varied from 236.7 to 917.0 g/L, and the procyanidin dimer, amentoflavone, methyl-biflavone, and digalloylquinic acid were dominant in all samples. The hydroxymethylfurfural (HMF) content of the herbal coffee varied between 0.01 and 0.39 mg/kg. According to a sensory analysis, the herbal coffee obtained from fruits roasted at 120 °C for 25 min was the most appreciated sample. In sum, this work shows that herbal coffee is non-caffeinated and is an alternative to regular coffee drinks derived from juniper berries roasted at lower temperatures and has more significant phenolic and antioxidant contents. It also has the potential to offer innovative and healthy alternatives to the food industry. Future research should focus on investigating how this herbal coffee can be positioned in the market and can influence consumer preference.

## 1. Introduction

Consumers’ demand for healthy products with different tastes and aromas and especially interest in herbal beverages produced with traditional methods have increased in the food industry in recent years. Various parts of plants (fruits, seeds, leaves, flowers, stems, roots, etc.) are used in the production of these beverages. These plant parts are treated with various cooking methods to add aroma and provide a brownish color like traditional Turkish coffee. Such beverages are called “herbal coffee” [1]. Significant changes occur in the phytochemical content and biological activity of herbal coffee obtained by the transformation of raw plant parts into the final product through various production stages [2,3].

Juniper berries, called “andiz” in Türkiye, are the fruits of a perennial tree (*Juniperus drupacea* L.) from the *Cupressacea* family [4]. Juniper berries are generally grown in Türkiye, Iran, Syria, and some Mediterranean countries and are particularly common in Central Anatolia, Southeast Anatolia, and the Aegean regions in Türkiye. Juniper berries are a versatile food ingredient with a rich history of use in different cultures. The berries are characterized by their aromatic flavor, making them a popular choice as a spice in a variety of dishes and beverages, particularly in the production of gin and other spirits [4]. Although juniper berry trees are naturally grown species, they are also cultivated commercially. Commercial production has been on an increasing trend to obtain more products to meet market demands [5]. Statistical data on their production is limited in comparison to other agricultural crops. The outer surface of the fruit is fleshy, and the fruit contains flat, hard-shelled seeds. It has a very rich potential of phenolic and antioxidant properties [6]. High levels of volatile oils (95.3–99.4%) have been detected in juniper berries, including α-pinene (22.8–63.4%) and limonene (10.7–51.1%) [7]. The fruits also have bioactive properties due to various phytochemicals, including flavonoids and phenolic substances [8]. These fruits are widely utilized in the regions where they are grown, generally by boiling and then drinking, in the treatment of lung and upper respiratory tract disorders and gastrointestinal and urological diseases [9]. Particularly, in recent years, these fruits have been utilized in the production of molasses (“pekmez” in Turkish). Satıl et al. [10], suggested that juniper berry molasses possessed beneficial properties similar to other fruit molasses, making it a viable option for both culinary and health applications. In another study by Konuk Takma [11], shell extracts of juniper berry fruits were utilized in biopackaging, and juniper berry molasses were appraised as a source of bioactive compounds. Moreover, according to Akbulut and Bilgiçli [12], juniper berry molasses could significantly alter the rheological and sensory properties of baked goods. Thus, juniper berry molasses could be an excellent natural sweetener and functional ingredient in various food products.

Another potential use of these fruits is the herbal coffee beverage obtained after roasting. It tastes similar to coffee but does not contain caffeine and gluten and has great potential for people who prefer coffee-flavored herbal drinks [13]. The roasting process increases the flavonoid content and antioxidant capacity of the fruits [14]. Juniper berries are safe for human consumption. They have high-value components that can be used in value-added food applications, including functional foods, nutraceuticals, pharmaceuticals, and medicinal ingredients [8].

Although there are studies on alternative beverages to coffee in the literature, there is no existing study on the production and bioactive properties of herbal coffee from juniper berries. This study aimed to evaluate the nutritional and bioactive properties of roasted juniper berries (“andiz” in Turkish) and to assess their usage as a new and innovative healthy herbal coffee with high quality and healthy properties. For this purpose, herbal coffee was obtained from roasted and powdered fruits grown in Türkiye in an oven at three different temperatures (120, 160, and 200 °C) and for four durations (10, 25, 32.5, and 55 min). The changes in color, dry matter, antioxidant activity, sugar composition (HPLC-RID), hydroxymethylfurfural (HMF) (HPLC-PDA), and phenolic compounds (LC-ESI-MS/MS) of the herbal coffee were investigated comprehensively based on these production parameters.

## 2. Materials and Methods

### 2.1. Juniper Berry Fruits

The fleshy parts of juniper berries (epidermis and mesocarp) usually account for about 50–70% of the total fruit weight, depending on factors such as maturity and environmental conditions. In this study, ripe juniper berry fruits (initially green with a reddish-brown color at ripening) (36 kg), used for the production of herbal coffee, were supplied from the forest areas located near the Sariaydın village of the Silifke district of Mersin Province, Türkiye (36°45′43.492″ N; 33°54′53.467″ E) in September 2022.

### 2.2. Chemicals

Methanol and ethanol (HPLC grade), formic acid, sodium carbonate, gallic acid, tyrosol, 4-hydroxy benzoic acid, vanillic acid, catechin, epicatechin, rutin, hydrochloric acid, ABTS [2,2-azinobis-(3-ethyl-benzothiazoline-6-sulfonic acid)], Trolox ((+/−)-6-hydroxy-2,5,7,8-tetramethyl-chroman-2-carboxylic acid), and DPPH (2,2-diphenyl-1-picrylhydrazylhydrazyl) were purchased from Merck Company (Merck KGaA, Darmstadt, Germany). Deionized water was used for the preparation of mobile phases in the HPLC analyses. Standard and other sensitive solutions were prepared daily.

### 2.3. Roasting and Preparation of Herbal Coffee

**Figure 1:**
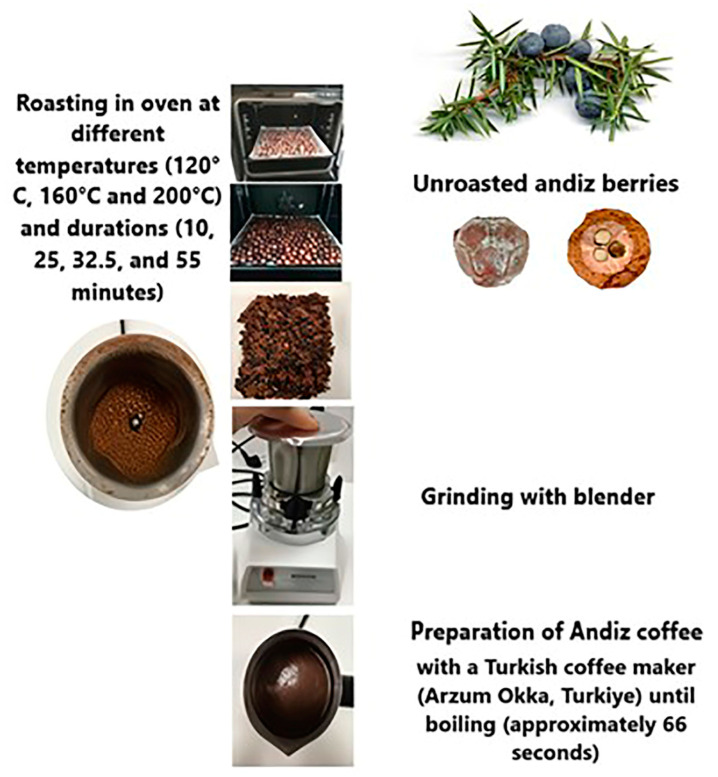
Production stages of herbal coffee samples from juniper berries roasted at three different temperatures and for four durations.

After ripe juniper berries were arranged on a tray, they were roasted in an oven at three different temperatures (120, 160, and 200 °C) and for four durations (10, 25, 32.5, and 55 min). These parameters were determined from the related literature [15,16,17]. Two kgs of fruits were roasted during each process (Figure 1). After roasting, the fleshy parts of the fruits were separated and ground with a waring blender (Waring Commercial Torrington, Model HGB2WTS3, Torrington, CT, USA) for about 1 min to a fine powder (Figure 1). In addition, raw fruit samples were ground and included in the analyses. Then, 8 g of the powdered fruits were added to 80 mL of water at room temperature. The herbal coffee samples were made with a Turkish coffee maker (Arzum Elektrikli Ev Aletleri San. ve Tic. A.Ş., Arzum Okka, Model OK004, Istanbul, Turkey) until boiling (approximately 66 s).

### 2.4. Color and Dry Matter Analyses

A chromameter (CM-5 Konica Minolta Inc., Osaka, Japan) was utilized to quantify the color of herbal coffee made from unroasted and roasted ground berries based on five parameters, including the L* (brightness), a* (redness/greenness), b* (yellowness/blueness), C* (chroma), and h (hue) color values of the raw juniper berry fruit and herbal coffee. A dry matter analysis of the herbal coffee was performed according to Cemeroglu [18], and the dry matter percentage was determined after being kept in an oven at 105 °C for 16 h.

### 2.5. Antioxidant Activity (AA) Analysis

The AA values of herbal coffee made from unroasted and roasted ground berries were determined according to two different methods: DPPH (2,2-diphenyl-1-picrylhydrazylhydrazyl) and ABTS (2,2-azinobis-(3-ethyl-benzothiazoline-6-sulfonic acid).

*In the DPPH method*, 0.1 mL of herbal coffee made from unroasted and roasted ground berries was mixed with 3.9 mL of a DPPH reagent, and after waiting in the dark for 30 min, absorbance values were determined with a spectrophotometer at a 515 nm wavelength (BMG Labtech, Spectrostar Nano, Ortenberg, Germany) [19]. Then, absorbance values were computed according to the Trolox standard slope chart (6.25–200.0 μmol/L), and the data are expressed in mM Trolox/kg dm.

*The ABTS method* was implemented according to Saafi et al. [20]. A total of 7.0 mM of ABTS was mixed with 2.45 mM of potassium bisulphate and was kept in the dark for 12–16 h. The solution was then diluted with a sodium acetate buffer (pH: 4.5) to obtain an absorbance of 0.70 ± 0.01 at a 734 nm wavelength in the spectrophotometer. Then, 2.98 mL of the prepared buffer was mixed into 20 μL of herbal coffee made from unroasted and roasted ground berries. After 10 min, absorbance values were measured at a 734 nm wavelength in the spectrophotometer. Absorbance data were computed based on the Trolox standard slope chart (10–100 μmol/L), and the values are stated in mM Trolox/kg dm.

### 2.6. Total Phenolic Content (TPC) Analysis

A TPC analysis was carried out according to Saafi et al. [20] with slight modifications. Before the analysis, 100 µL of herbal coffee made from unroasted and roasted ground berries was added to 0.5 mL of the Folin–Ciocalteu solution, and the mixture was kept for 5 min. Then, 6% sodium carbonate was added to this mixture, and 90 min later, absorbance values were read at 765 nm with a UV/VIS spectrophotometer (BMG Labtech, Spectrostar Nano, Ortenberg, Germany). The TPC values were then calculated with the calibration curve obtained from the absorbances of gallic acid standards prepared at different concentrations (6.25, 12.5, 25.0, 50.0, and 100.0 ppm) determined by the same method.

### 2.7. Hydroxymethylfurfural (HMF) Analysis

An HMF analysis was conducted according to the method of the International Federation of Fruit Juice Producers [21]. The HMF contents of herbal coffee made from unroasted and roasted ground berries were determined and quantified using an HPLC system (Shimadzu Corporation, Model Shimadzu Prominence-i LC-2030C, Kyoto, Japan) with a PDA detector and the Phenomenex Luna C18 column (250 × 4.6 mm, 5 µm) with a water/methanol mixture as the mobile phase (flow rate: 0.5 mL/min) [22]. The HMF standard was prepared at five different concentrations (between 1.56–100.0 ppm) to create the standard curve, and then, the HMF contents were quantified from the calibration curves in mg/kg dm.

### 2.8. Sugar Content Analysis

For the sugar analyses, herbal coffee samples, unroasted and roasted at three different temperatures and for four different durations, were centrifuged at 5500 rpm at 4 °C (Andreas Hettich GmbH Föhrenstr, Model Hettich Universal 320R, Tuttlingen, Germany) and the clear upper part was taken and filtered through 0.45 µm filters [23]. The extracts were then injected into the HPLC device (Shimadzu Prominence-i LC-2030C) with an RID detector to define and quantify the sugar contents. The Aminex HPX-87H (300 × 7.8 mm, Bio-Rad, Berkeley, CA, USA) column and 5 mM of sulfuric acid were utilized as the carrier phase with an isocritical system (flow rate: 0.5 mL/min). Calibration curves were created using sucrose, glucose, and fructose standards for the identification of compounds. The sugar contents of the extracts (mg/g DM) were determined using these curves.

### 2.9. Phenolic Profile Analysis

The phenolic compounds were analyzed according to Kelebek and Selli [24] with some modifications. One gram of herbal coffee made from unroasted and roasted ground berries was weighed into a centrifuge tube and was extracted with methanol–water (80:20, *v*/*v*). Then, these samples were centrifuged at 5500 rpm for 15 min at 4 °C. All samples were passed through a membrane filter with a pore size of 0.45 µm before injection. The phenolic compounds were determined by triple quadrupole mass spectroscopy (Model 6430, Agilent Tech., Palo Alto, CA, USA) HPLC with a diode array detector (DAD), an electrospray ionization source, and the Phenomenex Luna C-18 column (4.6 mm × 250 mm, 5 m). Mobile phase A consisted of water/formic acid (95:5, *v*/*v*), while phase B consisted of methanol/formic acid (95/5, *v*/*v*). The constituents were identified by the LC-MS/MS system in negative-ion mode using the following parameters: a capillary temperature and voltage of 400 °C and −3 V, a nebulizer gas flow of 1.75 L/min, a dissolution gas flow of 1 L/min, a spray voltage of 5 kV, and a scanning mass range of 100–2000 amu. Calibration curves of the standard phenolic compounds were utilized to determine the amount of each constituent. As it was not possible to obtain standard substances for all compounds, calibration curves prepared with comparable chemicals were used to determine the amounts of the constituents with no standards. Under the available chromatographic conditions, the limits of detection (LODs) and quantification (LOQs) were computed with signal/noise ratios (S/Ns) of approximately 3 and 10, respectively.

### 2.10. Sensory Analysis

The herbal coffee samples prepared with a coffee maker were subjected to a sensory analysis. Twelve herbal coffee samples produced unroasted and with different roasting parameters were tested by a group of six panelists (average age, 36; three women and three men), and in the sensory comparison, real coffee was prepared under the same conditions as the control group. Scoring was performed by marking the scores on a 10 cm scale (0, least liked and 10, most liked) based on 16 parameters (color, odor, taste, appearance, resin, foreign matters, roasted taste, acidity, astringency, fruitiness, aroma, sweetness, woody taste, bitterness, aftertaste, and general evaluation) [25].

### 2.11. Statistical Data Analysis

The experimental data of the herbal coffee were subjected to a variance analysis (ANOVA) at a 95% confidence level (*p* = 0.05) in the SPSS program (version 22, SPSS Inc., Chicago, IL, USA). The differences between the means were evaluated according to Duncan’s multiple comparison test. Moreover, the correlation matrix (Pearson correlation coefficients, r) was prepared to appraise multiple pairwise comparisons between the AA values, HMF contents, and phenolic compounds in the OriginPro software (version 9.8; OriginLab Corporation, Northampton, MA, USA).

## 3. Results

### 3.1. Dry Matter (DM) and Color Analyses Results

The DM contents of the unroasted juniper berry fruits and herbal coffee prepared from the roasted juniper berry fruits are given in Table 1. The mean DM amount of the unroasted juniper berries was found to be 83.67%, and it increased as a result of the roasting process. The highest DM content was observed in the herbal coffee roasted at 200 °C for 55 min (95.34%), while the lowest DM was found in the samples roasted at 120 °C for 10 min (84.95%). In another study, Özkan et al. [26] reported a DM value of 76.61% in *Juniperus drupacea* Labill fruits.

Color is an important quality parameter that draws attention by consumers and is important in terms of product acceptance [27]. It was observed that the mean L* (brightness) value of the unroasted (raw) juniper berry fruits was 38.72 (Table 1), and the L* values of the herbal coffees obtained after roasting at different temperatures and durations varied between 30.97 and 44.44 (Table 1). It was determined that the roasting temperature and duration caused a significant change in the L* value (*p* < 0.05). Increases were detected in the L* values of the herbal coffee prepared from the fruits roasted for 10 min at three different temperatures (120, 160, and 200 °C) compared to the unroasted fruits. It was determined that the roasting temperature and duration caused a significant change in the L* value (*p* < 0.05). Increases were detected in the L* values of the herbal coffee prepared from the fruits roasted for 10 min at three different temperatures (120, 160, and 200 °C) compared to the unroasted fruits. With an increased roasting duration, reductions were observed in the L* values of the herbal coffee roasted at 160 °C and 200 °C. During roasting, the water content of the fruit decreases. As the water evaporates, the color of the fruit becomes more intense. Initially, this may cause the L* value to increase as the loss of water causes the outer surface to reflect more light. In all samples, the darkness in color increased with a rise in roasting temperature and duration. Also, decreases in the a* values (redness) were detected in the herbal coffee with an increase in the degree of heat treatment and in duration. It was observed that the b* values of the herbal coffee varied within 3.71–18.56 and were positive in all samples (indicating a yellowish color). In addition, a decrease in the b* value occurred due to a rise in the roasting temperature and duration. It was found that the b* values decreased as the duration increased in the herbal coffee treated with a temperature of 120 and 160 °C. It was observed that the b* values of the herbal coffee prepared from the berries treated with 200 °C decreased (from 10 min to 32.5 min), but an increase occurred in the b* value for the heat treatment applied for a longer time (55 min) (Table 1). The C* (chroma) value refers to the saturation or intensity of a color, and higher values indicate a vivid and intense color, while lower values indicate more pastel or pale colors [28]. The highest mean C* value (23.58) was determined in the herbal coffee prepared from the berries after roasting at 120 °C for 25 min. h (hue) values indicate the type of color (red, blue, green, etc.) of a sample. It is usually measured in degrees, from 0° to 360° on the color wheel [28]. The highest mean h value was observed as 53.03 in the herbal coffee obtained from the fruits roasted at 120 °C for 25 min, indicating a color between yellow and green.

In some earlier studies conducted on juniper berry molasses (“pekmez” in Turkish), it was observed that the L* value varied between 8.30–12.01 [29] and 1.43–17.06 [30]. It is thought that a decrease in the L* value (darker color) is caused by high molecular weight compounds (melanoids) formed as a result of the Maillard reaction [31]. Ayseli [32] determined the L* value as 29.15, the a* value as 3.33, and the b* value as 2.20 in medium-roasted coffee and the L* value as 26.62, the a* value as 1.25, and the b* value as −0.40 in dark-roasted coffee samples. It has been mentioned in some studies that lower L* values and higher a* and b* values may be an indicator of an increase in the HMF content and may be due to the effect of burning sugar or the breakdown of vitamin C [33].

A general full factorial design was performed to reveal the main effects of temperature and duration factors and their interaction on some quality properties of herbal coffee based on 95% and 99% confidence levels. The results illustrate that the roasting temperature and time had a significant influence on the dry matter and color parameters of the herbal coffee (*p* ˂ 0.01) (Table 1).

**Table 1:** Color, dry matter (DM), antioxidant activity (AA-DPPH and AA-ABTS), total phenolic content (TPC), and hydroxymethylfurfural (HMF) values of the unroasted juniper berries and herbal coffee prepared from juniper berries roasted at three different temperatures and for four durations.

		120 °C				160 °C				200 °C						
Analyses	Unroasted Fruits	10 min	25 min	32.5 min	55 min	10 min	25 min	32.5 min	55 min	10 min	25 min	32.5 min	55 min	T	Tp	T × Tp
DM %	83.67 ± 0.18 ^a^	84.95 ± 0.24 ^b^	88.30 ± 0.14 ^de^	88.27 ± 0.04 ^de^	87.46 ± 0.38 ^c^	87.78 ± 0.04 ^cd^	88.96 ± 0.82 ^e^	91.65 ± 0.31 ^f^	94.32 ± 0.34 ^g^	88.40 ± 0.18 ^de^	91.77 ± 0.32 ^f^	94.87 ± 0.06 ^gh^	95.14 ± 0.29 ^h^	**	**	**
L*	38.72 ± 0.02 ^e^	38.93 ± 0.16 ^e^	44.44 ± 0.33 ^i^	40.34 ± 0.02 ^fg^	38.09 ± 0.40 ^e^	40.98 ± 0.08 ^gh^	40.04 ± 0.37 ^f^	39.95 ± 0.33 ^f^	33.79 ± 0.03 ^c^	41.47 ± 0.09 ^h^	35.96 ± 1.18 ^d^	32.10 ± 0.05 ^b^	30.97 ± 0.05 ^a^	**	**	**
a*	13.58 ± 0.01 ^f^	13.96 ± 0.55 ^fgh^	14.25 ± 0.32 ^ghi^	13.54 ± 0.02 ^f^	12.52 ± 0.13 ^e^	14.40 ± 0.02 ^hi^	14.15 ± 0.13 ^ghi^	13.84 ± 0.20 ^fg^	7.68 ± 0.04 ^c^	14.59 ± 0.03 ^i^	10.44 ± 0.14 ^d^	6.08 ± 0.02 ^b^	5.11 ± 0.14 ^a^	**	**	**
b*	15.71 ± 0.01 ^f^	16.81 ± 1.61 ^gh^	18.84 ± 0.04 ^j^	15.49 ± 0.02 ^f^	14.12 ± 0.55 ^e^	18.09 ± 0.04 ^ij^	17.20 ± 0.09 ^hi^	16.07 ± 0.34 ^fg^	8.23 ± 0.01 ^c^	18.51 ± 0.08 ^j^	12.23 ± 0.35 ^d^	6.56 ± 0.07 ^b^	5.31 ± 0.22 ^a^	**	**	**
C*	20.76 ± 0.01 ^fg^	21.88 ± 1.51 ^gh^	23.58 ± 0.11 ^i^	20.11 ± 0.67 ^f^	18.87 ± 0.50 ^e^	23.54 ± 0.66 ^i^	22.27 ± 0.15 ^h^	21.33 ± 0.40 ^gh^	11.26 ± 0.04 ^c^	23.57 ± 0.04 ^i^	16.08 ± 0.17 ^d^	8.94 ± 0.06 ^b^	7.39 ± 0.23 ^a^	**	**	**
h	49.20 ± 0.06 ^cd^	49.09 ± 0.93 ^cd^	53.03 ± 0.49 ^f^	49.69 ± 1.19 ^cd^	48.43 ± 0.81 ^bc^	51.77 ± 0.33 ^ef^	50.57 ± 0.12 ^de^	48.27 ± 0.63 ^bc^	46.98 ± 0.10 ^ab^	51.75 ± 0.16 ^ef^	49.51 ± 1.21 ^cd^	47.19 ± 0.22 ^ab^	46.78 ± 0.56 ^a^	**	**	**
DPPH	11.87 ± 0.05 ^d^	15.13 ± 0.38 ^f^	17.99 ± 0.03 ^h^	16.39 ± 0.02 ^g^	12.26 ± 0.07 ^d^	14.87 ± 0.06 ^f^	14.61 ± 0.14 ^f^	13.47 ± 1.13 ^e^	10.88 ± 0.05 ^c^	12.28 ± 0.19 ^d^	9.65 ± 0.07 ^b^	6.99 ± 0.02 ^a^	6.78 ± 0.04 ^a^	**	**	**
ABTS	15.99 ± 0.03 ^cd^	20.89 ± 0.03 ^g^	29.36 ± 0.07 ^i^	21.19 ± 0.02 ^g^	17.76 ± 0.34 ^ef^	23.36 ± 0.07 ^h^	21.63 ± 0.20 ^g^	18.53 ± 1.54 ^f^	15.12 ± 0.04 ^c^	16.72 ± 0.23 ^de^	13.72 ± 0.41 ^b^	11.44 ± 0.02 ^a^	11.04 ± 0.29 ^a^	**	**	**
TPC	1851.40 ± 1.41 ^d^	2351.70 ± 14.42 ^g^	2887.25 ± 1.06 ^i^	2494.00 ± 5.66 ^h^	1897.75 ± 46.03 ^de^	2139.50 ± 1.41 ^f^	2082.30 ± 6.08 ^f^	2004.40 ± 40.02 ^e^	1840.40 ± 2.40 ^cd^	1836.00 ± 25.03 ^cd^	1766.42 ± 8.37 ^bc^	1708.15 ± 7.71 ^b^	1543.05 ± 8.27 ^a^	**	**	**
HMF	nd	nd	nd	nd	nd	nd	nd	0.01 ± 0.00 ^a^	0.13 ± 0.00 ^b^	nd	0.14 ± 0.01 ^c^	0.27 ± 0.00 ^d^	0.39 ± 0.01 ^e^	**	**	**

a–j: different letters in the same row indicate significant differences (*p* < 0.05). DM, dry matter; DPPH, mM Trolox/L DW; ABTS, mM Trolox/L DW; TPC, mg GAE/L DW; HMF, mg/L DW; nd, not detected; T, time (min); Tp, temperature (°C); T × Tp, time × temperature; ** *p* ˂ 0.01

### 3.2. Results of Antioxidant Activity (AA) and Total Phenolic Content (TPC) Analysis

Antioxidants are defense systems that are responsible for preventing the damage caused by free radicals [34]. The caffeic, chlorogenic, coumaric, ferulic, nicotinic acids, caffeine, trigonelline, cafestol, and coffeeol available in coffee are compounds with high antioxidant potential [34,35]. In the present study, a decrease was found in the mean AA values of herbal coffee prepared from juniper berries roasted at the same temperature but with increasing durations (Table 1). Substantial differences were observed in the AA values of the herbal coffee (*p* ˂ 0.05).

It was observed that the AA values of the herbal coffee decreased with rising temperatures. It was observed that thermal decomposition as a result of the applied heat treatment had a negative effect on the AA values. The AA value based on the DPPH method in the unroasted fruits was 11.87 mM Trolox/L, while the highest AA was in the herbal coffee made from the juniper berries roasted at 120 °C for 25 min (17.99 mM Trolox/L), and the lowest AA was in the herbal coffee from the berries roasted at 200 °C for 55 min (6.78 mM Trolox/L). The AA determined by the ABTS method was 15.99 mM Trolox/L in the unroasted fruits, as the highest was in the herbal coffee prepared with fruits roasted at 120 °C for 25 min (29.36 mM Trolox/L), and the lowest was in the herbal coffee from the fruits roasted at 200 °C for 55 min (11.04 mM Trolox/L).

It was observed that the AA values of the herbal coffee computed by the DPPH and ABTS methods increased by roasting at 120 and 160 °C and for 25 min compared to unroasted fruits but decreased when the roasting temperature reached 200 °C (Table 1). Schouten et al. [36] examined the AA values of Arabica and Robusta coffees with five roasting degrees (light, light-medium, medium, medium-dark, and dark) according to the ABTS and DPPH methods, and they reported the AA values of the unroasted coffees as 3690 and 4400 mg Trolox/100 g, respectively. They quantified the AA values of the roasted samples as 5500 and 8150 mg Trolox/100 g (light), 6100 and 9080 mg Trolox/100 g (light-medium), 6770 and 9080 mg Trolox/100 g (medium), 7010 and 8910 mg Trolox/100 g (medium-dark), and 6680 and 8000 mg Trolox/100 g (dark), respectively. They stated that the AA values of the coffees increased up to a certain level and then decreased with an increase in the degree of roasting. This situation can be explained by both the formation and then decomposition of antioxidant compounds during roasting. At the beginning of roasting, phenols with a low molecular weight are released, and new constituents with antioxidant properties are formed as a result of Maillard reactions. However, as the roasting level increases, these newly formed compounds and existing antioxidants, such as chlorogenic acids, begin to decompose. Therefore, while an increase in the AA is observed at light and medium roasting degrees, the AA decreases in the darker roasting levels [36].

Table 1 shows the TPCs of the unroasted fruits and herbal coffee prepared from the roasted juniper berry fruits. Compared to the unroasted fruits, the TPCs of the herbal coffee increased as a result of roasting at 120 and 160 °C for 10 and 25 min, but a decrease was detected after 25 min, depending on the increasing duration. All roasting applications at 200 °C caused decreases in the TPC values of the herbal coffee (*p* < 0.05). While the TPC in the unroasted fruits was 1851.40 mg GAE/L, the highest TPC was found in the herbal coffee prepared from the fruits roasted at 120 °C for 25 min (2887.25 mg GAE/L), and the lowest was determined in the herbal coffee prepared from the fruits roasted at 200 °C for 55 min (1543.05 mg GAE/L). Similar studies examining the effects of roasting on *Juniperus drupacea* fruits are quite limited. Özkan et al. [26], determined the TPC in *J. drupacea* fruits as 25,000 mg GAE/g. Additionally, Alasalvar et al. [37] found that the amount of condensed tannins and gallic acid in Turkish hazelnuts reduced significantly with roasting. Schmitzer et al. [38] also reported drops in the amounts of phenolics, especially catechin derivatives, as a result of roasting.

A general full factorial design was performed to reveal the effects of temperature and duration factors and their interaction with the AA and TPC levels of the herbal coffee based on 95% and 99% significance levels (Table 1). The data show that the roasting temperatures and durations had a significant effect on the AA and TPC levels of the herbal coffee (*p* ˂ 0.01).

Thermal processing of plant-based products, including roasting, can lead to the degradation of sensitive phytochemicals, especially phenolic compounds known for their antioxidant properties. Akbulut and Akbulut [8] emphasized that the most abundant phenolic substance in *J. drupacea* fruits was protocatechuic acid, which is sensitive to thermal degradation. High temperatures can accelerate the degradation of these constituents, causing a significant decrease in both TPC and AA values [39]. Xiong et al. [40] stated that some thermal treatments can increase certain antioxidant properties, but excessive heat leads to a decrease in overall AA values due to the destruction of phenolic substances. In addition, the Maillard reaction, occurring during roasting, can also affect the antioxidant properties of plant-based materials. This reaction can form new compounds that may exhibit antioxidant properties or consume existing antioxidants, leading to a decrease in their AA levels [15]. Thus, the balance between the creation of useful Maillard reaction compounds and the degradation of phenolics is very important [40]. In addition, specific roasting conditions such as temperature and duration play a critical role in influencing the final antioxidant profile. Gümral et al. [41] reported that some volatile constituents in *Juniperus* species can increase antioxidant enzyme activities, but these benefits may not compensate for the losses resulting from thermal degradation at higher temperatures. This is consistent with the findings from other studies showing that optimum roasting conditions are required to maximize antioxidant retention [42]. The decrease in the AA and TPC levels in the *J. drupacea* fruits with an increasing temperature and infusion time in the current study may be attributed to the thermal degradation of sensitive phenolic compounds, potential adverse effects of the Maillard reaction, and the applied thermal treatment conditions. The findings obtained in the present study parallel those of previous studies on the effects of heat treatments on the phytochemical integrity of plant-based materials.

### 3.3. Results of Hydroxymethylfurfural (HMF) Analysis

**Figure 2:**
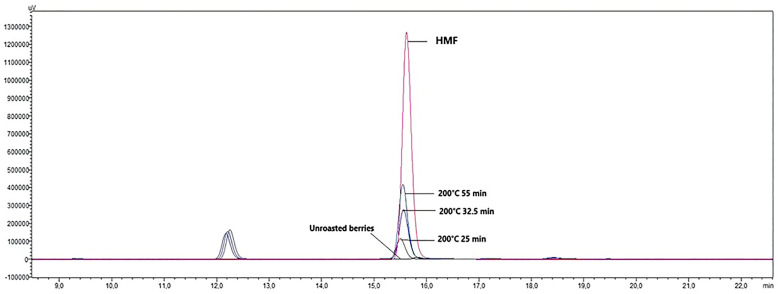
Chromatogram of the hydroxymethylfurfural (HMF) of the herbal coffee from roasted juniper berries (uV vs. retention time (min)).

The Maillard reaction occurs among free amino acids, proteins (peptides), free amino groups, and reducing sugars or lipid oxidation products in foods, and one of the reaction products is hydroxymethylfurfural (HMF) [43,44]. The chromatogram of the HMF compound is shown in Figure 2. In the current study, HMF was not detected in the herbal coffee obtained from the juniper berries roasted at 120 °C at all four durations, while it was formed at higher temperatures and longer durations (Table 1). In particular, the HMF content of the herbal coffee obtained from the berries roasted at 200 °C increased relatively with an increase in the roasting duration (*p* < 0.05). The highest HMF amount (0.39 mg/L) was obtained as a result of 200 °C and 55 min of roasting (Figure 2). It has been reported in previous studies that the type of coffee beans, the roasting temperature, and duration affect the quantity of HMF, and the rate of HMF formation increases as the roasting temperature and duration increase, while very high temperatures will cause a structural HMF breakdown [17].

Numerous compounds are formed from various reactions due to high temperatures applied when roasting coffee, and they contribute to taste and aroma but can also have harmful effects on human health [45]. Seninde et al. [31] reported that constituents such as furan (2-methyl furan, 2-acetyl furan, and 5-furfural) and HMF (5-hydroxymethyl-2-furfural), which define the aroma and flavor characteristics of coffee, are formed as a result of the collapse of sugars and proteins during roasting. In another study, Murkovic and Bornik [45] obtained an amount of HMF ranging from 0.3 to 1.9 mg/g in commercial roasted coffee. High temperatures in heat treatments accelerate the rate of carbohydrate degradation.

Shapla et al. [46] stated that HMF concentrations increase significantly at temperatures exceeding 120 °C. Laukaleja and Krūma [47] reported that as roasting temperatures rise, the degradation of carbohydrates and subsequent HMF formation accelerate, and melanoidin from chlorogenic acids, which are the precursors of HMF, is formed. Longer roasting times in the roasting of *J. drupacea* fruits allow more extensive caramelization and Maillard reactions, leading to an increase in HMF quantity. It is also thought that the degradation of organic acids facilitates HMF production, creating an acidic environment during roasting [48]. In another study, Park et al. [17] reported that HMF levels in roasted coffee beans are considerably affected by the roasting temperature and duration, with maximum concentrations being observed at higher temperatures and longer times. Similarly, studies on other plant-based products have shown a direct correlation between heat treatment conditions and HMF levels [49]. In the current study, the amounts of HMF detected in the juniper berry herbal coffee are lower than the stated levels in the literature, and this is thought to be due to the low protein content of these fruits [29,33].

### 3.4. Results of Sugar Content Analysis

**Figure 3:**
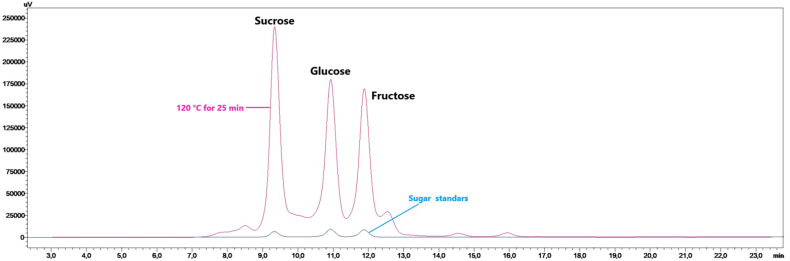
HPLC chromatogram of the sugar compounds of herbal coffee from juniper berries (uV vs. retention time (min)).

Three sugar compounds, sucrose, glucose, and fructose, were analyzed in the juniper berry herbal coffee (Table 2). The HPLC chromatogram of these sugars is given in Figure 3. Sucrose and glucose were found to be dominant in all herbal coffee samples. The highest sucrose and glucose contents were determined as 19.83 and 18.82 g/L in the herbal coffee roasted at 120 °C for 25 min, respectively (*p* < 0.05). The total sugar (TS) amounts of the herbal coffee varied within 13.55–51.42 g/L, while the highest quantity (51.42 g/L) was detected in the herbal coffee roasted at 120 °C for 25 min (Table 2). The TS level decreased as a result of the rise in roasting temperature and duration. In the roasting applied at 160 °C, the highest TS level (40.56 g/L) was determined in the herbal coffee obtained from the berries roasted for 32.5 min. In the roasting process performed at 200 °C, the TS amount decreased regardless of the duration.

The influence of roasting on the sugar content is a crucial determinant of food quality. The Maillard reaction that occurs during roasting involves a reaction between reducing sugars and amino acids, causing the formation of some compounds, including melanoidins [50]. This reaction consumes free sugars and reduces their total concentration [51]. Taha and Matthäus [52] emphasized that roasting can lead to significant changes in the sugar profile, and some sugars can be converted into other compounds or can be lost due to thermal degradation. In addition, roasting parameters such as temperature and duration play a critical role in these changes. Chung et al. [51] reported that the amount of sugar in corn grains decrease considerably with the rise in roasting duration and temperature, and there are significant losses in sugar content when the grains are exposed to heat for a long time. In sum, the decrease in the sugar content of *J. drupacea* fruits during roasting can be explained by the reactions in which sugars enter through thermal degradation and Maillard reactions. The sugar content is affected by roasting conditions; hence, the roasting temperature and duration must be carefully controlled to preserve beneficial compounds.

A general full factorial design was performed to reveal the effects of temperature and duration factors and their interaction with the sugar contents of herbal coffee in the present study based on 95% and 99% significance levels. The results indicate that the roasting temperature and duration had an important effect on the glucose, sucrose, fructose, and total sugar contents (*p* ˂ 0.01) (Table 2). 

**Table 2:** Sugar contents (g/L) of the unroasted juniper berries and herbal coffee obtained from juniper berries roasted at three different temperatures and for four durations.

		120 °C				160 °C				200 °C						
Sugar Contents	Unroasted Fruits	10 min	25 min	32.5 min	55 min	10 min	25 min	32.5 min	55 min	10 min	25 min	32.5 min	55 min	T	Tp	T × Tp
Sucrose	5.01 ± 0.05 ^b^	12.31 ± 0.05 ^d^	19.83 ± 0.13 ^h^	13.95 ± 0.11 ^e^	15.10 ± 1.24 ^fg^	12.78 ± 0.06 ^d^	14.61 ± 0.49 ^ef^	15.70 ± 0.64 ^g^	9.45 ± 0.18 ^c^	12.49 ± 0.39 ^d^	9.46 ± 0.27 ^c^	5.77 ± 0.09 ^b^	3.68 ± 0.05 ^a^	**	**	**
Glucose	4.91 ± 0.03 ^a^	12.69 ± 0.30 ^d^	18.82 ± 0.22 ^g^	14.08 ± 0.13 ^ef^	14.99 ± 1.46 ^f^	12.97 ± 0.11 ^de^	14.03 ± 0.44 ^ef^	14.49 ± 0.66 ^f^	12.56 ± 0.48 ^d^	12.25 ± 0.26 ^d^	10.30 ± 0.32 ^c^	7.22 ± 0.05 ^b^	5.61 ± 0.04 ^a^	**	**	**
Fructose	3.64 ± 0.02 ^a^	9.59 ± 1.18 ^efg^	12.78 ± 0.31 ^h^	9.10 ± 0.03 ^ef^	10.25 ± 0.47 ^fg^	8.66 ± 0.23 ^de^	10.03 ± 0.87 ^fg^	10.38 ± 0.57 ^g^	9.14 ± 0.11 ^ef^	7.74 ± 0.33 ^cd^	6.78 ± 0.49 ^c^	5.65 ± 0.23 ^b^	4.47 ± 0.16 ^a^	**	**	**
Total sugar	*13.55 ± 0.11 ^a^*	*34.59 ± 1.52 ^ef^*	*51.42 ± 0.04 ^i^*	*37.12 ± 0.27 ^fg^*	*40.33 ± 3.17 ^h^*	*34.41 ± 0.40 ^ef^*	*38.68 ± 1.79 ^gh^*	*40.56 ± 1.87 ^h^*	*31.15 ± 0.55 ^d^*	*32.47 ± 0.45 ^de^*	*26.54 ± 1.08 ^c^*	*18.63 ± 0.37 ^b^*	*13.75 ± 0.24 ^a^*	**	**	**

a–i: different letters in the same row indicate significant differences (*p* < 0.05). T, time (min); Tp, temperature (°C); T × Tp, time × temperature; ** *p* ˂ 0.01.

### 3.5. Results of Phenolic Compound Analysis

**Figure 4:**
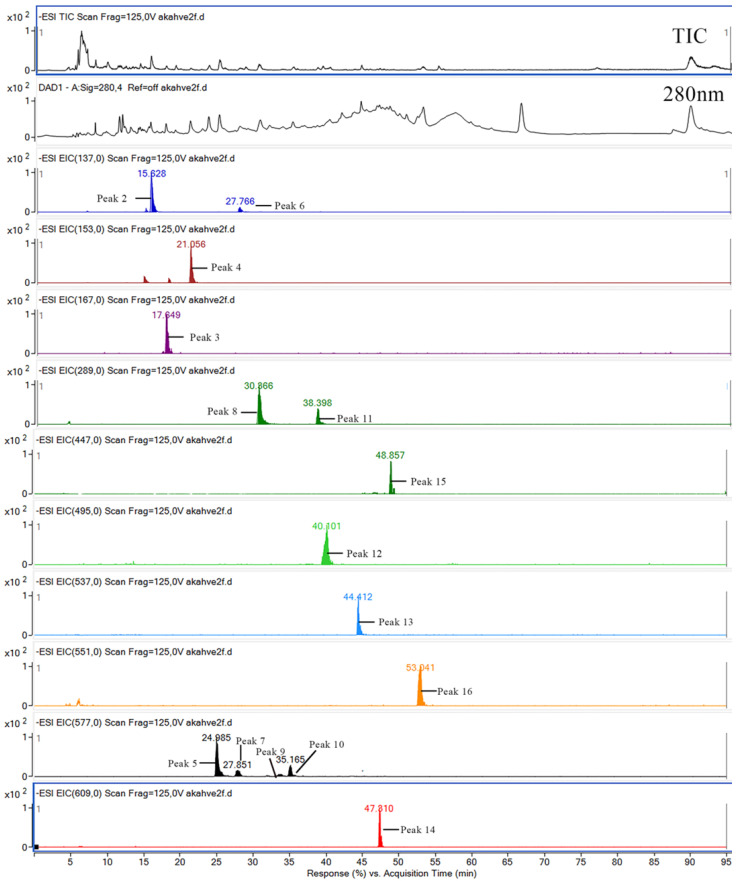
LC-ESI-DAD-MS/MS chromatograms of herbal coffee from roasted juniper berries. The peaks correspond to the compounds in Table 3. (TIC, total ion chromatogram).

A total of 16 phenolic substances, including gallic acid, tyrosol, 4-hydroxy benzoic acid, vanillic acid, dihydroxy benzoic acid, catechin, epicatechin, digalloylquinic acid, amentoflavone, methyl-biflavone, rutin, quercetin-rhamnosyl, and four additional procyanidin dimers, were identified and quantified in the juniper berry herbal coffee (Table 3). The chromatograms of the identified compounds recorded at 280 and 320 nm are illustrated in Figure 4. Substantial differences were found between the phenolic contents of the herbal coffee (*p* < 0.05). The total phenolic contents (TPCs) varied between 236.7 and 917.0 g/L, and the highest value was found for the roasting at 120 °C for 25 min. In the unroasted fruits, the dominant phenolics were the procyanidin dimer (108.11 mg/L), amentoflavone (27.89 mg/L), and digalloylquinic acid (13.31 mg/L). In the herbal coffee, on the other hand, the dominant phenolics were the procyanidin dimer, amentoflavone, methyl-biflavone, and digalloylquinic acid, respectively, and their concentrations were higher in the roasting process at 120 °C for 25 min, but their quantities decreased depending on the higher temperature and duration (Table 3). It was observed that the roasting process increased the amount of phenolics compared to the unroasted berries. Tyrosol, p-hydroxy benzoic acid, dihydroxy benzoic acid hexoside, vanillic acid, and amentoflavone were identified in different juniper berry molasses (“pekmez” in Turkish) samples in earlier work [33]. Gallic acid, tyrosol, p-hydroxy benzoic, vanillic acid, dihydroxy benzoic acid, and amentoflavone were reported in juniper berry extracts by Ereli [33], with p-hydroxy benzoic (8.32–11.48 µg/g) and vanillic acids (8.26–10.36 µg/g) being the dominant phenolics.

**Figure 5:**
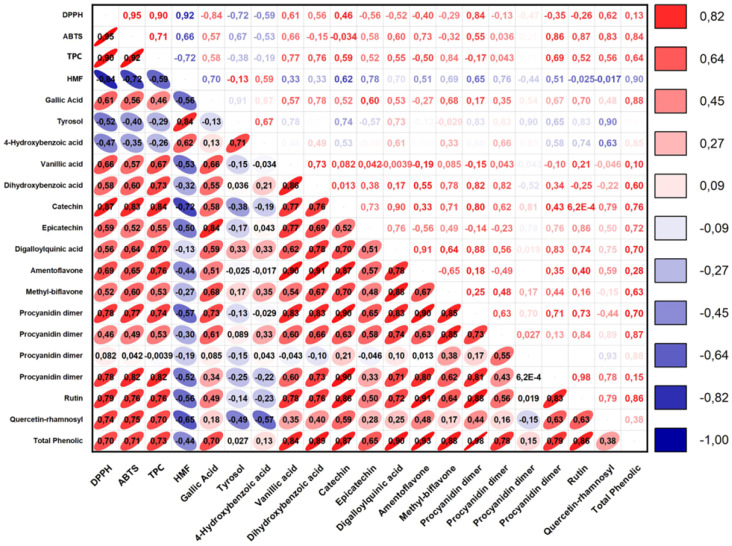
Correlation matrix of the antioxidant activity (DPPH and ABTS), total phenolic content (TPC), hydroxymethylfurfural (HMF), and phenolic compounds of herbal coffee obtained from roasted juniper berries (*p* ≤ 0.05).

The correlation data among the antioxidant capacity and phenolic compounds of the herbal coffee are shown in Figure 4. A moderate negative correlation was observed between the tyrosol and AA values determined by the DPPH (r = −0.52) and ABTS (r = −0.40) methods. Positive moderate–strong correlations were observed between vanillic acid and DPPH (r = 0.66), ABTS (r = 0.57), and TPC (r = 0.67) (Figure 5). A strong and positive correlation was seen between the procyanidin dimer and TPCs (r = 0.98). It was also observed that the correlation between the digalloylquinic acid content and TPC amount was also strong and positive (r = 0.70). Furthermore, a moderate correlation was determined for the gallic acid contents. In general, substantial differences were observed among the compounds (*p* < 0.05). Juniper berry fruits contain various phenolic acids and flavonoids, with the most dominant being protocatechuic acid [4]. In another investigation, a total of 18 phenolics were determined in these fruits, with protocatechuic acid being quantified as 77.1 μg/g in fruits and 251.6 μg/g in molasses [8]. Other important phenolics were reported as gallic acid, chlorogenic acid, and tyrosol, contributing to the antioxidant properties of the fruits [26]. In another study by Miceli et al. [4], gallic acid, tyrosol, amentoflavone, and protocatechic acid were identified in the methanol extract of juniper berry fruits. Additionally, Özdemir et al. [6] reported the presence of gallic acid (9.6–286.0 mg/kg) in juniper berry molasses.

A general full factorial design was performed to reveal the effects of temperature and duration factors and their interaction with the phenolic compounds and TPCs based on 95% and 99% significance levels. The roasting temperature and duration have a significant effect on most of the phenolic substances (*p* ˂ 0.01) (Table 3). However, temperature was found to have no significant effect on the phenolic compound of “rutin”. Also, the time × temperature interaction term had no significant effect on the quercetin-rhamnosyl compound (*p* ˃ 0.05).

Phenolic constituents identified in the juniper berry fruits, including gallic acid, tyrosol, 4-hydroxybenzoic acid, *p*-hydroxybenzoic acid, vanillic acid, dihydroxybenzoic acid, catechin, epicatechin, digalloylquinic acid, amentoflavone, methyl-biflavone, rutin, quercetin-rhamnosyl, and procyanidin dimer, have beneficial health effects [26]. Most of these phenolics have a significant antioxidant capacity.

**Table 3:** Phenolic compound concentrations (mg/L) of the unroasted juniper berries and herbal coffee obtained from juniper berries roasted at three different temperatures and for four durations.

						120 °C				160 °C				200 °C						
Peak No	Compounds	RT	Precursor Ion (m/z) M^−^	Product Ions (m/z)	Unroasted Fruits	10 min	25 min	32.5 min	55 min	10 min	25 min	32.5 min	55 min	10 min	25 min	32.5 min	55 min	T	Tm	T × Tm
1	Gallic acid	11.71	169	125	3.41 ± 0.13 ^d^	6.01 ± 0.22 ^e^	9.17 ± 0.09 ^h^	7.39 ± 0.06 ^g^	6.56 ± 0.13 ^f^	6.58 ± 0.04 ^f^	9.09 ± 0.05 ^h^	11.59 ± 0.13 ^j^	10.81 ± 0.35 ^i^	3.44 ± 0.16 ^d^	2.82 ± 0.08 ^c^	2.23 ± 0.02 ^b^	1.17 ± 0.06 ^a^	**	**	**
2	Tyrosol	15.63	137	119	8.31 ± 0.14 ^a^	24.20 ± 0.45 ^e^	26.30 ± 1.00 ^f^	22.92 ± 0.12 ^e^	20.53 ± 1.09 ^d^	18.25 ± 0.02 ^c^	22.77 ± 0.02 ^e^	26.76 ± 0.78 ^f^	30.88 ± 0.69 ^g^	12.79 ± 0.71 ^b^	23.68 ± 0.96 ^e^	35.02 ± 0.55 ^h^	55.95 ± 0.90 ^i^	**	**	**
3	Vanillic acid	17.65	167	152, 123, 108	6.03 ± 0.17 ^ab^	21.91 ± 0.57 ^efg^	23.98 ± 7.50 ^fgh^	28.86 ± 0.19 ^h^	24.66 ± 1.83 ^gh^	9.74 ± 0.02 ^bc^	14.36 ± 0.09 ^cd^	18.58 ± 0.37 ^de^	19.22 ± 0.48 ^def^	10.23 ± 1.01 ^bc^	9.56 ± 0.62 ^bc^	8.71 ± 0.47 ^ab^	4.04 ± 0.11 ^a^	**	*	**
4	Dihydroxy benzoic acid	21.06	153	109	8.26 ± 0.09 ^a^	29.85 ± 0.24 ^de^	53.85 ± 0.39 ^i^	47.84 ± 0.27 ^h^	41.63 ± 1.03 ^g^	23.54 ± 0.68 ^c^	28.89 ± 0.18 ^de^	31.23 ± 3.21 ^ef^	33.41 ± 0.61 ^f^	22.06 ± 0.89 ^c^	23.89 ± 1.12 ^c^	28.17 ± 0.34 ^d^	17.01 ± 0.21 ^b^	**	**	**
5	Procyanidin dimer	24.98	577	289, 245	108.11 ± 0.58 ^a^	354.06 ± 3.16 ^h^	411.80 ± 0.76 ^j^	374.23 ± 0.47 ^i^	349.75 ± 1.21 ^h^	331.03 ± 0.15 ^g^	330.92 ± 1.30 ^g^	334.20 ± 2.33 ^g^	312.26 ± 0.37 ^f^	272.71 ± 3.76 ^e^	231.59 ± 0.83 ^d^	189.32 ± 0.47 ^c^	175.01 ± 5.45 ^b^	**	**	**
6	4-Hydroxy benzoic acid	27.66	137	93	4.22 ± 0.05 ^a^	9.51 ± 0.25 ^c^	16.20 ± 0.28 ^g^	10.79 ± 0.33 ^d^	12.20 ± 0.14 ^e^	8.90 ± 0.03 ^c^	14.92 ± 0.22 ^f^	19.89 ± 1.02 ^i^	18.85 ± 0.30 ^h^	7.68 ± 0.04 ^b^	16.52 ± 0.74 ^g^	26.93 ± 0.71 ^j^	18.79 ± 0.63 ^h^	**	**	**
7	Procyanidin dimer	27.85	577	289, 245	6.05 ± 0.81 ^a^	13.56 ± 0.58 ^f^	16.47 ± 0.11 ^g^	10.80 ± 0.42 ^c^	10.97 ± 0.60 ^cd^	8.95 ± 0.10 ^b^	11.64 ± 0.50 ^cde^	14.04 ± 0.68 ^f^	12.02 ± 0.08 ^de^	12.39 ± 0.38 ^e^	11.17 ± 0.58 ^cd^	10.54 ± 0.69 ^c^	8.82 ± 0.36 ^b^	**	**	**
8	Catechin	30.21	289	245, 205, 179	8.95 ± 0.40 ^b^	29.11 ± 0.79 ^h^	33.66 ± 0.51 ^i^	34.76 ± 0.49 ^i^	24.35 ± 1.15 ^f^	26.74 ± 0.56 ^g^	26.62 ± 0.22 ^g^	25.10 ± 0.97 ^f^	16.02 ± 0.46 ^c^	22.27 ± 0.75 ^e^	18.26 ± 0.55 ^d^	15.06 ± 0.71 ^c^	4.48 ± 0.02 ^a^	**	**	**
9	Procyanidin dimer	33.77	577	289, 245	1.81 ± 0.03 ^ab^	4.88 ± 0.28 ^gh^	3.14 ± 0.01 ^de^	1.73 ± 0.04 ^a^	1.82 ± 0.08 ^ab^	2.69 ± 0.05 ^bcd^	3.42 ± 0.02 ^def^	4.08 ± 0.01 ^fg^	2.94 ± 0.05 ^cd^	5.65 ± 1.36 ^h^	3.88 ± 0.11 ^ef^	3.22 ± 0.01 ^def^	2.11 ± 0.02 ^abc^	**	**	**
10	Procyanidin dimer	35.16	577	289, 245	3.92 ± 0.06 ^a^	9.56 ± 0.33 ^fg^	13.88 ± 0.24 ^i^	13.51 ± 0.06 ^i^	9.58 ± 0.56 ^fg^	12.67 ± 0.08 ^h^	10.04 ± 0.13 ^g^	7.37 ± 0.25 ^de^	6.35 ± 0.03 ^c^	8.99 ± 0.39 ^f^	7.50 ± 0.43 ^e^	6.86 ± 0.05 ^cd^	5.08 ± 0.06 ^b^	**	**	**
11	Epicatechin	38.39	289–245	245, 205, 179	2.66 ± 0.07 ^cd^	2.51 ± 0.54 ^bcd^	5.61 ± 0.16 ^f^	4.86 ± 0.08 ^f^	3.77 ± 0.14 ^e^	1.80 ± 0.02 ^abc^	3.08 ± 0.12 ^de^	4.91 ± 1.06 ^f^	5.63 ± 0.36 ^f^	2.55 ± 0.57 ^bcd^	1.68 ± 0.09 ^ab^	1.35 ± 0.01 ^a^	0.98 ± 0.09 ^a^	**	**	**
12	Digalloylquinic acid	40.10	495	343, 191	13.31 ± 0.48 ^a^	38.02 ± 0.28 ^g^	49.63 ± 0.82 ^i^	41.54 ± 4.20 ^h^	29.50 ± 1.90 ^cd^	36.21 ± 1.01 f^g^	37.02 ± 0.13 ^fg^	37.89 ± 0.33 ^g^	35.23 ± 0.47 ^f^	26.85 ± 0.98 ^b^	27.88 ± 0.91 ^bc^	30.25 ± 0.91 ^d^	33.11 ± 0.88 ^e^	**	**	**
13	Amentoflavone	44.41	537	493, 443, 417, 374	27.89 ± 1.09 ^a^	109.08 ± 0.52 ^i^	120.58 ± 1.08 ^j^	134.26 ± 1.64 ^k^	109.83 ± 2.62 ^i^	75.59 ± 2.64 ^f^	82.83 ± 0.24 ^g^	87.74 ± 2.18 ^h^	73.62 ± 0.75 ^f^	70.64 ± 0.83 ^e^	64.98 ± 1.22 ^d^	61.81 ± 0.27 ^c^	56.82 ± 0.51 ^b^	**	**	**
14	Rutin	47.31	609	301, 273, 257, 179, 151	0.93 ± 0.04 ^a^	2.27 ± 0.02 ^cde^	2.63 ± 0.02 ^e^	2.71 ± 0.02 ^e^	2.35 ± 0.43 ^de^	1.98 ± 0.06 ^cd^	2.02 ± 0.03 ^cd^	1.92 ± 0.20 ^cd^	1.39 ± 0.02 ^ab^	1.80 ± 0.06 ^bc^	1.33 ± 0.09 ^ab^	0.98 ± 0.05 ^a^	1.42 ± 0.55 ^ab^	**	ns	**
15	Quercetin-rhamnosyl	48.85	447	301, 151	1.29 ± 0.06 ^ef^	1.17 ± 0.21 ^cde^	1.70 ± 0.72 ^e^	1.32 ± 0.02 ^ef^	1.34 ± 0.07 ^ef^	1.25 ± 0.04 ^def^	1.12 ± 0.03 ^de^	0.89 ± 0.16 ^abcde^	0.51 ± 0.02 ^a^	1.03 ± 0.05 ^bcde^	0.76 ± 0.07 ^abcd^	0.54 ± 0.03 ^ab^	0.67 ± 0.03 ^abc^	**	*	ns
16	Methyl-biflavone	53.04	551	-	31.541.00 ^a^	98.63 ± 0.67 ^g^	128.37 ± 0.78 ^k^	88.88 ± 0.32 ^e^	90.70 ± 1.16 ^e^	101.73 ± 0.86 ^h^	109.69 ± 0.44 ^i^	115.26 ± 1.62 ^j^	95.68 ± 0.89 ^f^	88.98 ± 1.48 ^e^	84.86 ± 1.07 ^d^	80.68 ± 0.76 ^c^	77.06 ± 1.06 ^b^	**	**	**
	*Total*				*236.7 ± 5.20 ^a^*	*754.3 ± 8.59 ^i^*	*917.0 ± 2.59 ^k^*	*826.4 ± 2.25 ^j^*	*739.6 ± 9.81 ^h^*	*667.7 ± 3.37 ^f^*	*708.4 ± 0.70 ^g^*	*741.5 ± 6.22 ^hi^*	*674.8 ± 5.94 ^f^*	*570.1 ± 0.46 ^e^*	*530.4 ± 9.47 ^d^*	*501.7 ± 6.05 ^c^*	*462.5 ± 6.85 ^b^*	**	**	**

a–k: different letters in the same row indicate significant differences (*p* < 0.05). RT, retention time (min); T, time (min); Tp, temperature (°C); T × Tp, time × temperature; ** *p* ˂ 0.01; * *p* ˂ 0.05; ns, not significant.

Duskaev et al. [53] reported that vanillic acid scavenges free radicals and increases antioxidant enzyme activity, such as that of glutathione peroxidase and superoxide dismutase. Tyrosol is also known for its antioxidant properties and protective benefits against oxidative stress [54]. The antioxidant capacity of phenolics plays a crucial role in reducing oxidative damage, which is associated with numerous chronic diseases [55]. Various phenolic substances, including vanillic acid and catechin, exhibit anti-inflammatory effects. Pawar et al. [56] reported that vanillic acid inhibits the synthesis of pro-inflammatory cytokines such as TNF-α and IL-6, decreasing inflammation. Similarly, catechin and epicatechin have been associated with inflammatory modulation, which may contribute to their potential to prevent inflammatory diseases [57]. Vanillic acid also inhibits the growth of various bacteria, including multidrug-resistant strains [58]. This antimicrobial activity is of great importance, especially in the context of food preservation and therapeutic applications against infections. Gong et al. [59] emphasized that vanillic acid inhibits tumor growth and angiogenesis in some cancer cell lines. Similarly, catechin and epicatechin can induce apoptosis in cancer cells and can inhibit tumor progression [57]. Phenolics such as rutin and quercetin-rhamnosyl have cardiovascular protective effects and can improve endothelial function, lower blood pressure, and inhibit platelet aggregation [60]. These significant effects are attributed to the ability of the compounds to increase nitric oxide production and reduce oxidative stress in vascular tissues [61]. Additionally, phenolics such as rutin and quercetin help in managing diabetes and obesity by acting on metabolic pathways, including improved glucose metabolism and lipid profiles [60]. Amentoflavone and methyl-biflavone have been associated with neuroprotective effects, helping to protect neuronal cells from oxidative stress and inflammation, which are critical factors in neurodegenerative diseases [62]. Phenolic compounds available in juniper berries offer various health benefits, including anti-inflammatory, antioxidant, antimicrobial, cardiovascular, anti-cancer, neuroprotective, and metabolic effects. These properties underscore the potential of these constituents to promote health and prevent diseases [62].

Roasting results in not only degradation but also the release of phenolic substances. Thermal treatments can degrade sensitive phenolic structures, leading to a decrease in their overall concentration. Protocatechuic acid and other phenolic acids are known to be thermally unstable, and their levels decrease considerably when exposed to high temperatures for long periods [12,63]. This degradation is generally attributed to the thermal polymerization and oxidation processes that occur during roasting [63]. Thermal treatments can cause the polymerization, oxidation, or degradation of phenolics, leading to a shift in the phenolic profile [64]. This degradation is particularly pronounced for compounds such as chlorogenic acid, which is thermally unstable [65]. Similarly, Zhou et al. [66] reported that roasting may lead to the loss of some phenolics. Conversely, suitable roasting conditions may facilitate the release of bound phenolics from their matrix, potentially increasing the quantity of free phenolics. Chbani et al. [16] emphasized that roasting may liberate phenolics from their esterified or bound forms, which may lead to a temporary increase in their availability. However, as roasting continues, the balance shifts towards degradation, resulting in an overall decrease in phenolic content. This dual effect leads a crucial role in understanding the changes of phenolic profiles during roasting [67].

Roasting conditions such as temperature and duration play a critical role in influencing the extent of changes in the phenolic profile. Studies have shown that a proper roasting process can increase the extraction of phenolics, but excessive heat or long roasting times can lead to significant losses [52,66]. Bubueanu et al. [65] stated that roasting can significantly alter the phenolic composition, especially for thermally sensitive compounds. In the case of juniper berry fruits, optimum roasting conditions need to be applied to maximize the retention of beneficial phenolics while minimizing their degradation. The data obtained in the present study and the findings in the literature reveal that suitable roasting conditions are quite important for conserving beneficial compounds.

### 3.6. Results of Sensory Analysis 

**Figure 6:**
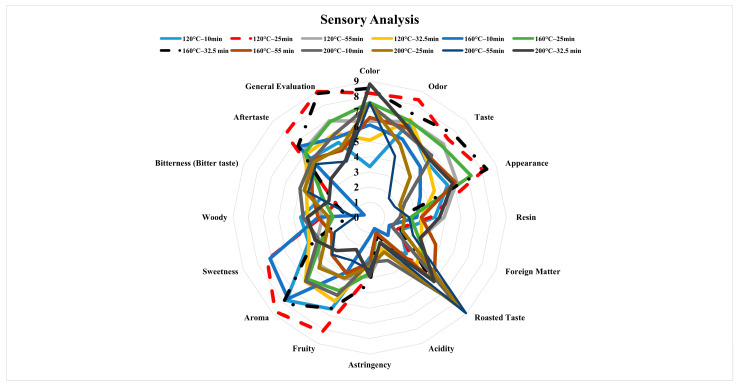
Sensory analysis results of herbal coffee from juniper berries roasted at three different temperatures and for four durations.

The taste of a food item is one of the crucial factors that affect consumer preference [68]. The concept of flavor is described as the combination of taste and smell, and the first impression of food by consumers is formed by appearance, texture, and flavor [25]. In the current study, 16 evaluation parameters were used in the sensory evaluations (color, odor, taste, appearance, resin, foreign matter/impurities, roasted taste, acidity, astringency, fruitiness, sweetness, woody taste, bitterness, aftertaste, and overall evaluation). A spider-web diagram of the analysis results is given in Figure 6. According to the obtained data, the herbal coffee made from fruits roasted at 120 °C and 25 min was the most liked sample, followed by the sample at 160 °C and 32.5 min (Figure 6). It was observed that the bitter taste increased with a rise in the roasting temperature and duration. In addition, the juniper berries roasted at a higher temperature (200 °C) and for a longer duration (55 min) received lower scores because of burning that occurred during roasting. The roasting temperature and duration also significantly changed the profiles of aromatic compounds formed during roasting.

## 4. Conclusions

In this study, herbal coffee obtained from juniper berry fruits roasted at three different temperatures and for four different durations were prepared, and their color; dry matter (DM); antioxidant activity (AA, DPPH, and ABTS); total phenolic content (TPC); sugar composition; hydroxymethylfurfural (HMF) content; and phenolic profiles were investigated. It was found that the temperature and duration applied in the roasting process caused significant changes in the color of the herbal coffee (L*, a*, and b* values) compared to the unroasted juniper berries. The TPC values of the samples were negatively affected by the rise in the roasting temperature and duration. The highest TPC and AA values were determined in the herbal coffee obtained from the berries roasted at 120 °C for 25 min. The HMF content could not be detected in the unroasted fruits, while the HMF contents of the herbal coffee samples varied between 0.0 and 0.39 mg/kg DM. Sucrose was the dominant sugar in the unroasted fruits, as sucrose and glucose were dominant in the herbal coffee. A total of 16 phenolic compounds were identified in the unroasted fruits and herbal coffee samples. The TPC values varied between 236.7 and 917.0 g/L in all samples, and the procyanidin dimer, amentoflavone, methyl-biflavone, and digalloylquinic acid were the most dominant phenolics. A strong positive correlation was observed between the AA and TPC contents. In general, the TPC amounts decreased with increasing roasting temperatures and durations. According to the sensory analysis, the herbal coffee produced from the fruits roasted at 120 °C for 25 min were the most appreciated sample, followed by the sample roasted at 160 °C and for 32.5 min in terms of aftertaste, sweetness, aroma, fruitiness, appearance, odor, and general evaluation parameters. In sum, it was found that the herbal coffee obtained from roasted juniper berries had good potential in terms of phenolic compounds and antioxidant capacity. In future research, a more comprehensive examination of the impacts of different roasting methods and fruit varieties may contribute to improving the nutritional value of these herbal coffees and developing new strategies to meet consumer preferences. Moreover, juniper berry herbal coffee offers an attractive option, especially for those who drink non-caffeinated coffee and for health-conscious consumers. As research continues to reveal the health benefits of non-caffeinated coffee, it is clear that juniper berry herbal coffee can play an important role in a healthy diet.

## Data Availability

The original contributions presented in the study are included in the article, further inquiries can be directed to the corresponding author.
